# Inflammatory markers in prepubertal children and their associations with abdominal fat

**DOI:** 10.1016/j.jped.2024.03.015

**Published:** 2024-05-08

**Authors:** Letícia G. de Queiroz, Paulo F. Collett-Solberg, Maria das Graças C. de Souza, Nádia Cristina P. Rodrigues, Alexandra M. Monteiro, Cristiane de S. Mendes, Fernanda M. Gazolla, Cecília L. de Oliveira, Eliete Bouskela, Maria Cristina C. Kuschnir, Isabel R. Madeira

**Affiliations:** a Universidade do Estado do Rio de Janeiro, Faculdade de Ciências Médicas, Programa de Pós-Graduação em Ciências Médicas, Rio de Janeiro, RJ, Brazil; b Universidade do Estado do Rio de Janeiro, Faculdade de Ciências Médicas, Departamento de Medicina Interna, Disciplina de Endocrinologia, Rio de Janeiro, RJ, Brazil; c Universidade do Estado do Rio de Janeiro, Centro Biomédico, Laboratório de Pesquisas Clínicas e Experimentais em Biologia Vascular, Rio de Janeiro, RJ, Brazil; d Universidade do Estado do Rio de Janeiro, Instituto de Medicina Social Hésio Cordeiro, Rio de Janeiro, RJ, Brazil; e Universidade do Estado do Rio de Janeiro, Faculdade de Ciências Médicas, Rio de Janeiro, RJ, Brazil; f Universidade do Estado do Rio de Janeiro, Hospital Universitário Pedro Ernesto, Laboratório de Hormônios, Rio de Janeiro, RJ, Brazil; g Universidade do Estado do Rio de Janeiro, Hospital Universitário Pedro Ernesto, Unidade de Ensino de Endocrinologia e Metabologia, Rio de Janeiro, RJ, Brazil; h Universidade do Estado do Rio de Janeiro, Instituto de Nutrição, Departamento de Nutrição Aplicada, Rio de Janeiro, RJ, Brazil; i Universidade do Estado do Rio de Janeiro, Faculdade de Ciências Médicas, Departamento de Pediatria, Rio de Janeiro, RJ, Brazil

**Keywords:** Pediatric obesity, Abdominal fat, Inflammation

## Abstract

**Objective:**

To evaluate the association between inflammatory markers and abdominal fat assessed by ultrasound in prepubertal children with and without excess weight.

**Methods:**

A cross-sectional study involving 241 prepubertal children, 156 with obesity, 37 with overweight, and 48 with normal weight, aged five to ten years, who were followed at a research unit on Childhood Obesity from a teaching hospital belonging to a public health system. The concentration of interleukin-6, tumor necrosis factor-α and C-reactive protein were assessed and regression analyses, considering outcome variables such as abdominal wall and intra-abdominal fat thickness measured by ultrasound, were performed.

**Results:**

The findings highlighted an association between abdominal fat and inflammatory markers, even in children at this young age group. Subcutaneous fat showed a stronger association with inflammatory biomarkers compared to intra-abdominal fat when performing logistic regression, with a positive association between tumor necrosis factor-α and abdominal wall thickness equal to or greater than the 75th percentile in adjusted logistic regression (OR: 18.12; CI 95 %: 1.57: 209.55).

**Conclusions:**

Abdominal wall fat, in contrast to what is often observed in adults, appears to have a greater impact on chronic inflammation related to excessive weight in very young children.

## Introduction

The prevalence of childhood obesity has increased significantly in recent decades.^1^ A 2019 Brazilian report indicated that one-third of children between five and nine years of age had excessive weight.^2^ The 2023 World Obesity Atlas suggests that global expenditure caused by excess weight, directly and indirectly, will exceed 4 trillion dollars in 2035, which corresponds to around 3% of global gross domestic product.^3^ Previous studies have demonstrated an association between childhood excess weight and cardiovascular risk,^4^ and that the atherosclerotic process begins in the early years of life.^5^ A longitudinal study that evaluated the associations between cardiovascular biomarkers and metabolic syndrome during puberty showed adolescent individuals with metabolic syndrome already had several altered markers when prepubertal, such as body mass index (BMI) Z score, waist circumference, insulin, homeostasis model assessment for insulin resistance (HOMA-IR) and leptin, unlike those without metabolic syndrome, highlighting the importance of the prepubertal period in relation to future comorbidities.^6^ Thus, identifying cardiovascular risk markers in children with excess weight becomes essential for future cardiovascular disease prevention. Among the non-classical markers, inflammatory biomarkers stand out.

Obesity has been associated with a state of low-grade chronic systemic inflammation, with interleukin-6 (IL-6), interleukin-1, interleukin-8, and tumor necrosis factor-α (TNF-α) playing an important role and resulting in elevations of acute-phase proteins such as C-reactive protein (CRP).^7^

*In vitro* and *in vivo* studies have already shown the ability of IL-6 to induce insulin resistance. High levels of IL-6 are considered risk factors for metabolic syndrome as they cause lipid and vascular changes that accompany insulin resistance.^8^ IL-6 promotes a reduction in the activity of lipoprotein lipase, and in turn, generates an increase in the flow of fatty acids to the liver.^9^

TNF-α also has a positive correlation with obesity and modulates leptin,^10^ as well as stimulates the production of IL-6 and CRP.^8^ It is a multifunctional inflammatory cytokine secreted by adipocytes, neutrophils, lymphocytes and macrophages, and affects lipid metabolism, coagulation, endothelial function and generates insulin resistance.^11^

CRP is an acute phase reagent produced by the liver and identified as a sensitive marker of inflammation, also acting as a mediator of cardiometabolic disease.^12^ A previous study has also demonstrated the strong association of elevated CRP with increased adipose tissue in humans, even in childhood, and with insulin resistance.^8^ Chronic elevation of CRP plays an important role in the arteriosclerosis process and the development of cardiovascular diseases by impairing endothelial function resulting in the thickening of the intima media.^13^

There are different types of body fat deposits. The main ones are abdominal subcutaneous fat, visceral fat, gluteofemoral subcutaneous fat and intramuscular fat. In adults, abdominal fat, especially visceral fat, is more closely associated with inflammatory markers,^14^ and consequently with increased cardiovascular risk, as it releases more inflammatory cytokines per gram of tissue.^8^

However, there are few studies focusing on body fat distribution in children and its link with inflammation, especially in prepubertals. This age group is of particular interest due to the increased opportunity for preventive interventions.

Furthermore, excess body adiposity can be assessed through anthropometric measurements and imaging tests. Imaging tests can better assess the distribution of body fat, including computed tomography, magnetic resonance imaging, dual-energy X-ray absorptiometry and ultrasound. Ultrasonography has the advantage of being a quick, lower-cost exam and does not involve ionizing radiation. Existing data in the literature point to promising perspectives for the use of ultrasound in determining body composition in different areas and clinical situations, with ultrasound proving to be a method with potential practical application in the clinic.^15^

## Methods

The purpose of this study was to examine the association between abdominal wall thickness (AWT) and intra-abdominal fat thickness (IAFT) measured by ultrasound and the inflammatory markers, IL-6, CRP, TNF-α in prepubertal children.

This cross-sectional study involved a convenience sample of 241 children: 156 with obesity, 37 with overweight, and 48 with normal weight, based on the World Health Organization definition,^16^ all recruited from the Pediatrics Ambulatory of the same institution between 2008 and 2023. During this period, 507 individuals were initially recruited, of which 241 met the inclusion criteria and had the data studied in this research. The study was approved by the institution's Research Ethics Committee, CAAE protocol number: 51457721.5.0000.5259.

Children included in the study were between five and ten years old, either with excess weight or of normal weight, prepubertal, and whose guardians have agreed to participate in the research by signing the written informed consent form after the project was explained to them. Puberty assessment was conducted by pediatric endocrinologists, defining prepubertal patients using Tanner 1 stage.^17^ Exclusion criteria included the presence of comorbidities and or the regular use of medication that could influence the assessment of studied parameters.

Weight was assessed using a Filizola scale (Filizola, São Paulo, SP, Brazil) with a resolution of 100 g, and height was measured using a wall-mounted stadiometer (Tonelli, Criciúma, SC, Brazil) with a resolution of 1 mm. Children were weighed and measured using light clothing and without shoes.

Waist circumference (WC) was measured on undressed children, midway between the lower border of the last rib and the iliac crest,^18^ taken at the end of a normal exhalation using a Gulik measuring tape (Cardiomed, Curitiba, PR, Brazil). The waist circumference to height ratio (WC/Ht) ratio was calculated by dividing the WC by the patient's height.

AWT and IAFT were measured via ultrasound by an experienced radiologist. For measurements, the child laid supine with the transducer placed about 2 cm above the umbilical scar, all readings were taken in the axial plane without transducer pressure on the abdomen. AWT was defined as the distance between skin and linea alba, and IAFT as the distance from the posterior face of linea alba to the anterior wall of the aorta. Both measurements utilized an Aplio XG ultrasound machine, model SSA-790A (Toshiba Medical Systems, Tokyo, Japan) with a 3.5 MHz convex transducer for IAFT measurement and a 12 MHz linear transducer for AWT. No prior preparation for the exam was required. The choice of ultrasound as the imaging method was due to its non-ionizing radiation, lower cost, quick execution, no need for sedation, and its good correlations with computed tomography.^19^^,^^20^ Based on these measurements children were divided into groups. The 75th percentile (P75) was used as a cutoff point. This cutoff was chosen aiming to evaluate the last quartile of AWT and IAFT measurements, seeking the association between the highest values of these measurements and the markers studied. The group of children with AWT equal to or greater than the P75 was denominated AWT ≥ P75 and the group with children with IAFT equal to or greater than the P75 was denominated IAFT ≥ P75.

For assessment of inflammatory and metabolic markers in serum, children needed to be free from any acute disease at the time of collection and were asked to fast for 12 h. All evaluations were taken at the institution's laboratories.

Circulating serum levels of IL-6 were determined using an immunoassay method with Roche kits (Roche, Basel, Switzerland) on serum.

Serum concentrations of CRP, TNF-α, adiponectin, and leptin were evaluated using commercially available enzyme-linked immunosorbent assay (ELISA) kits (R&D Systems (Minneapolis, MN, USA)) and performed according to directions provided by the kit's manufacturer.

For CRP, results > 7.163 µg/mL were excluded for exceeding the last standard of the curve, and for TNF-α, values < 0.156 pg/mL were discarded for being below the curve's lowest value.

Fasting glucose, high-density lipoprotein cholesterol (HDL-c), and triglycerides (TG) blood concentrations were evaluated on Konelab equipment with a BT 3000 Winer kit (Winterlab, Rosario, Santa Fe, Argentina), utilizing the GOD-PAP (oxidase) enzymatic method for glucose and TG and the non-precipitation colorimetric enzymatic method for HDL-c.

Blood insulin levels were assessed using a kit employing electrochemiluminescence method (Roche, Basel, Switzerland) and processed on Cobas e601 equipment. HOMA-IR was subsequently calculated.^21^

Data collected were analyzed using the R-Project 4.2.1 software (Free Software Foundation, Boston, MA, USA).

Continuous variables studied were: age (years), WC/Ht, AWT and IAFT (mm), IL-6 (pg/mL), CRP (µg/mL), TNF-α (pg/mL), HDL-c (mg/dL), TG (mg/dL), HOMA-IR, leptin (ng/dL), and adiponectin (µg/mL). Categorical variables included: gender, AWT ≥ P75, and IAFT ≥ P75. Age, gender, WC/Ht, HDL-c, TG, HOMA-IR, leptin, and adiponectin were also assessed as potential confounders.

Initially, it was observed whether the distribution of continuous variables followed a normal distribution or not using the Shapiro-Wilk test. The appropriate method of description and analysis was chosen for continuous and categorical variables, as well as for association analyses.

Medians, first quartile and third quartile of continuous variables were then calculated, as well as the absolute and relative frequencies of categorical variables in the descriptive analysis.

Considering that AWT and IAFT did not present a normal distribution, non-parametric regression models were used to evaluate the association between these outcome variables and the other variables. Median-based linear models (Kendall-Theil Sen Siegel) were used with the calculation of the regression coefficient and 95% confidence interval to evaluate the simple association of predictors in relation to outcomes.

Simple and adjusted logistic regression models were also used, with AWT ≥ P75 and IAFT ≥ P75 as separate outcome variables. For adjusted logistic regression analysis, the following independent variables were considered: age, WC/Ht, IL-6, CRP, TNF-α, leptin, adiponectin, TG, HDL-c, and HOMA-IR. Statistical significance was considered when p-value < 0.05.

## Results

All continuous variables studied showed non-normal distribution. Therefore, the median and its quartiles were considered for the descriptive analysis. The frequencies of categorical variables were also calculated. Table 1 presents the medians of the entire group and Table 2 presents the demographic characteristics of the entire sample and of each sample of individuals according to their weight.

**Table 1:** Descriptive analysis of outcome variables, anthropometric, metabolic variables and inflammatory markers/ adipokines in the total sample.

Variables	Median (1st – 3rd quartiles)
**Anthropometric**	
WC/ Ht	0.56 (0.49 - 0.61)
**Metabolic**	
HDL-c (mg/dL)	44.98 (38.00 - 51.00)
TG (mg/dL)	85.00 (61.00 - 122.00)
HOMA-IR	1.82 (1.00 - 3.20)
**Inflammatory markers/ Adipokines**	
IL-6 (pg/mL)	2.47 (1.50 - 3.91)
CRP (µg/mL)	1.84 (0.90 - 3.63)
TNF-α (pg/mL)	0.92 (0.70 - 1.24)
Adiponectin (µg/mL)	9.09 (6.04 - 12.56)
Leptin (ng/dL)	13.73 (5.45 - 23.74)
**Outcomes (US analysis)**	
AWT (mm)	18.90 (10.6 - 24.2)
IAFT (mm)	38.40 (28.9 - 47.7)

AWT, abdominal wall thickness; IAFT, intra-abdominal fat thickness; WC/Ht, waist circumference to height ratio; HDL-c, high-density lipoprotein cholesterol; TG, triglycerides; HOMA-IR, homeostasis model assessment for insulin resistance; IL-6, interleukin-6; CRP, C-reactive protein; TNF-α, tumor necrosis factor-α.

**Table 2:** Demographic profile of the total sample.

Variables	Obese	Overweight	Normal weight	Total sample
**Median of age (years)** **(1st - 3rd quartiles)**	8.08 (6.73 - 8.98)	8.16 (7.10 - 9.75)	8.00 (7.00 - 8.82)	8.00 (6.83 - 9.06)
**Sex% (N)**	Female: 44.87 (70)	Female: 54.05 (20)	Female: 52.08 (25)	Female: 47.72 (115)
	Male: 55.13 (86)	Male: 45.95 (17)	Male: 47.92 (23)	Male: 52.28 (126)

In the simple analysis with AWT as the outcome variable, only sex did not show a significant result. When IAFT was taken as the outcome variable, all independent variables exhibited statistical significance as demonstrated in Table 3.

**Table 3:** Simple analysis of predictors in relation to abdominal wall thickness and to intra-abdominal fat thickness.^a^

Variables	AWT			IAFT		
	Regression coefficient	CI 95 %	P- value	Regression coefficient	CI 95 %	P-value
**Demographic**						
Age	2.09	1.65 : 2.47	0.0001	2.03	1.41 : 2.57	0.0001
Sex						
Female	0			0		
Male	0.40	−0.38 : 0.29	0.93	3.40	1.30 : 5.05	0.001
**Anthropometric**						
WC/ Ht	97.88	94.25 : 102.11	0.0001	110.74	103.13 : 117.47	0.0001
**Metabolic**						
HDL-c	−0.21	−0.25 : −0.13	0.0001	−0.12	−0.20 : −0.23	0.0001
TG	0.11	0.09 : 0.11	0.0001	0.11	0.09 : 0.14	0.0001
HOMA-IR	3.47	3.37 : 4.00	0.0001	3.64	3.32 : 4.22	0.0001
**Inflammatory markers/ Adipokines**						
IL-6	1.89	1.50 : 2.36	0.0001	2.14	1.85 : 3.02	0.0001
CRP	2.56	2.29 : 2.92	0.0001	2.65	2.05 : 3.10	0.0001
TNF-α	10.69	8.96 : 13.56	0.0001	7.62	5.34 : 11.06	0.0001
Adiponectin	−0.87	−1.00 : −0.75	0.0001	−1.14	−1.34 : - 1.03	0.0001
Leptin	0.69	0.65 : 0.74	0.0001	0.60	0.53 : 0.66	0.0001

AWT, abdominal wall thickness; IAFT, intra-abdominal fat thickness; CI, confidence interval; WC/Ht, waist circumference to height ratio; HDL-c, high-density lipoprotein cholesterol; TG, triglycerides; HOMA-IR, homeostasis model assessment for insulin resistance; IL-6, interleukin-6; CRP, C-reactive protein; TNF-α, tumor necrosis factor-α.

a Median-based linear models were used for simple analysis.

In both simple analysis models with either AWT or IAFT as the dependent variable, a higher regression coefficient was observed for inflammatory biomarkers, especially for TNF-α, compared to metabolic data and other adipokines.

For IL-6 and CRP, a higher coefficient was observed in the analysis with IAFT as the dependent variable, and for TNF-α that was found with AWT, as shown in Table 3.

A total of 51 individuals presented AWT ≥ P75 and 57 individuals presented IAFT ≥ P75. 24 individuals presented only AWT ≥ P75, 30 individuals presented only IAFT ≥ P75 and 27 individuals presented AWT and IAFT ≥ P75.

In the simple logistic regression with the outcome variable being AWT ≥ P75, there was a positive association with WC/Ht, HOMA-IR and TNF-α, and a negative one with adiponectin. Among the evaluated markers, TNF-α exhibited the highest Odds Ratio (OR). In adjusted logistic regression, both WC/Ht and TNF-α maintained a positive association, with the OR for TNF-α being even higher. Age also showed a positive association.

For the simple logistic regression with the outcome variable being IAFT ≥ P75, both WC/Ht and TNF-α showed a positive association and adiponectin a negative one. In the adjusted logistic regression, only WC/Ht showed a positive association, as Table 4 demonstrates.

**Table 4:** Crude and adjusted effect of the predictors and abdominal wall thickness greater than or equal to 75th percentile and intra-abdominal fat thickness greater than or equal to 75th percentile.^a^^,^^b^

	AWT					IAFT				
Variables	Crude OR	CI 95 %	Adjusted OR	CI 95 %	p-value	Crude OR	CI 95 %	AdjustedOR	CI 95 %	p-value
**Demographic**										
Age	1.22	0.87:1.71	1.80	1.03:3.15	0.039	1.15	0.81:1.62	1.43	0.89:2.29	0.141
**Anthropometric**										
WC/Ht	1.55×10^11^	4.00×10^5^ : 5.98×10^16^	4.99×10^10^	1,70×10^2^: 1.47×10^19^	0.013	5.82×10^8^	8.74×10^3^ : 3.89×10^13^	3.38×10^11^	2.29×10^4^ : 5.00×10^18^	0.002
**Metabolic**										
HDL- c	0.97	0.92:1.02	0.97	0.90:1.05	0.458	0.99	0.94:1.04	1.01	0.95:1.08	0.702
TG	1.01	1.00:1.02	1.00	0.99:1.01	0.580	1.01	1.00:1.01	1.00	0.99:1.01	0.955
HOMA-IR	1.83	1.24:2.70	1.32	0.76:2.29	0.327	1.28	0.94:1.74	0.84	0.51:1.41	0.418
**Inflammatory markers/ Adipokines**										
IL- 6	1.00	0.83:1.21	1.21	0.88:1.66	0.241	1.00	0.83:1.21	1.07	0.81:1.41	0.641
CRP	1.31	0.96:1.78	0.99	0.59:1.66	0.963	1.19	0.87:1.62	0.84	0.51:1.40	0.501
TNF- α	14.77	2.81:77.51	18.12	1.57:209.55	0.020	4.66	1.07:20.36	3.77	0.60:23.50	0.156
Adiponectin	0.76	0.64:0.91	0.91	0.72:2.29	0.449	0.85	0.74:0.99	0.98	0.83:1.16	0.787
Leptin	1.04	1.00:1.07	0.97	0.9:1.05	0.440	1.02	0.99:1.05	0.98	0.92:1.04	0.459

AWT, abdominal wall thickness; IAFT, intra-abdominal fat thickness; OR, odds-ratio; CI, confidence Interval; WC/Ht, waist circumference to height ratio; HDL-c, high-density lipoprotein cholesterol; TG, triglycerides; HOMA-IR, homeostasis model assessment for insulin resistance; IL-6, interleukin-6; CRP, C-reactive protein; TNF-α, tumor necrosis factor α.

a At the adjusted model, the following independent variables were considered: age, WC/Ht, IL-6, CRP, TNF-α, leptin, adiponectin, TG, HDL-c and HOMA-IR.

b Logistic regression models were used for the analysis.

## Discussion

This study demonstrated an association between increased abdominal fat measured by ultrasonography and inflammation in prepubertal children. It was observed that increased abdominal fat was more associated with inflammatory markers than with metabolic parameters, except for HOMA-IR.

The relationship between increased AWT and inflammation was also observed in both simple and adjusted logistic regressions. The simple model displayed a stronger positive association between increased AWT and TNF-α (OR: 14.77) than the association between increased IAFT and TNF-α (OR: 4.66). In the adjusted analysis, the association between the outcome variable IAFT ≥ P75 and TNF-α lost statistical significance, emphasizing once more that, in this sample of children, subcutaneous fat is more related to inflammation than intra-abdominal fat.

Several studies have evaluated inflammation and abdominal fat in children and adolescents, revealing a relationship between increased central adiposity and inflammatory markers.^22^^,^^23^ Multiple studies have also explored abdominal and non-abdominal fat deposits. However, few studies have investigated inflammation and its relationship with abdominal fat deposits measured by imaging tests, in individuals both with and without excessive weight, particularly in prepubertal children, with conflicting results, as it is going to be discussed.

Pou and colleagues found that, in adults, excess adiposity was associated with systemic inflammation. In the Framingham study involving adult participants, inflammatory markers and their relationships with subcutaneous and visceral fat were assessed using computed tomography. The results indicated that increases in subcutaneous and visceral fat were similarly related to CRP (Pearson correlation coefficients: 0.45 and 0.47 in women and 0.30 and 0.33 in men), IL-6 (Pearson: 0.23 and 0.23), and TNF-α (Pearson: 0.05 and 0.06).^24^

A review by Hermsdorf and Monteiro concluded that TNF-α, IL-6, and CRP are secreted in larger amounts by visceral fat, especially when analyzing adult obese individuals. This phenomenon was related to the total increased fat accumulation in visceral tissue and not the adipocyte size itself. Abdominal subcutaneous tissue showed intermediate cytokine expression compared to visceral and gluteo-femoral subcutaneous tissues.^25^

In a study of obese adolescents using dual-energy X-ray absorptiometry for fat measurement, a relationship was found between increased visceral and subcutaneous fats and CRP (Spearman correlation coefficients: 0.45 and 0.47, respectively).^26^

Moss and colleagues studied individuals between 12 and 23 years of age with severe obesity and evaluated the relationship between intra-abdominal fat measured by ultrasound and metabolic and inflammatory markers. A positive association was observed between IL-6 and intra-abdominal fat in both females and males (regression coefficients: 0.31 and 0.45, respectively) and between CRP and intra-abdominal fat (regression coefficient: 0.38),^27^ indicating that the association of intra-abdominal fat with inflammatory markers is present after puberty.

The present study found different results when comparing the association of intra-abdominal and subcutaneous fat to the inflammatory markers, probably due to the younger age of the studied population.

In this study, the cutoff point for AWT and IAFT, used in the logistic regression, was P75, as there are no cutoff points established in the literature for these measurements in prepubertal children. A study that evaluated IAFT and its relationship with metabolic syndrome in adolescents demonstrated that the 95th percentile showed the highest strength of association with the occurrence of metabolic syndrome and a higher specificity value.^28^ However, this article did not evaluate inflammatory markers.

In a previous study within the same group of researchers, in which both AWT and IAFT were assessed by ultrasound in prepubertal children, it was observed that there was a greater increase in AWT and not in IAFT with the increase in BMI Z-score, which may explain the greater role of this fat in inflammation.^29^ With more accumulation of IAFT over a lifetime, it would start having a greater influence on inflammation than the former one as individuals age.

Similar to results found in the present study, another one with a population of prepubertal children with and without excess weight, analyzed the relationship between abdominal fat measured by magnetic resonance imaging and inflammatory cytokines, and found a relationship between subcutaneous abdominal fat and TNF-α (regression coefficient: 0.3895, p: 0.0367), without any relationship with visceral fat.^30^

This corroborates the concept that in younger individuals the association of subcutaneous fat with inflammatory markers is stronger than with intra-abdominal one. Probably after puberty that changes.

The main limitation of this study was the absence of cutoff points for abdominal fat measurements assessed by ultrasound and for the inflammatory cytokines themselves, which prevents further conclusions from being drawn from the findings. However, the measurements assessed through ultrasound were calculated using a device operated by a radiologist with extensive experience in ultrasound.

Another limitation was that the study sample was of convenience, therefore, its results must be applied or extrapolated with caution to other groups of children.

Few studies evaluate inflammation and abdominal fat assessed by imaging tests exclusively in prepubertal children. The current one uniquely evaluates inflammatory biomarkers in a sample of children, with and without excess weight, in the prepubertal stage, an age range of particular interest for its high potential for prevention and treatment of obesity and its comorbidities, and their associations with abdominal fat measured by ultrasound, a more feasible test in pediatrics.

The importance of this study is that it indicates that in prepubertal children there is a stronger association between inflammatory markers and AWT than when compared to IAFT, a result that is opposite to the one found in adults and adolescents.

The importance of puberty and pubertal hormones in the effects of fat distribution on inflammatory markers should be better investigated.

## Declaration of generative AI and AI-assisted technologies in the writing process

During the preparation of this work, the author used Chat Generative Pre-Trained Transformer 4.0 in order to improve language. After using this tool, the author reviewed and edited the content as needed and took full responsibility for the content of the publication.

## Financial support

Financial support (APQ1) from Fundação Carlos Chagas Filho de Amparo à Pesquisa do Estado do Rio de Janeiro Foundation (FAPERJ), protocol numbers E-26/202.866/2015 and E-26/201.014/2022.

## Authors’ contributions

Letícia G. de Queiroz: This author has contributed to the conception and design of the study, acquisition, analysis and interpretation of data, drafting the article, revising it critically for important intellectual content and to the final approval of the version to be submitted.

Paulo F. Collett-Solberg: This author contributed to the acquisition and interpretation of data, revising it critically for important intellectual content and to the final approval of the version to be submitted.

Maria das Graças C. de Souza: This author contributed to the acquisition and analysis of data, revising it critically for important intellectual content, and to the final approval of the version to be submitted.

Nádia Cristina P. Rodrigues: This author contributed to the analysis and interpretation of data, revising it critically for important intellectual content and to the final approval of the version to be submitted.

Alexandra M. Monteiro: This author contributed to the acquisition and analysis of data.

Cristiane de S. Mendes: This author contributed to the acquisition of data, revising it critically for important intellectual content, and to the final approval of the version to be submitted.

Fernanda M. Gazolla: This author contributed to the acquisition of data, revising it critically for important intellectual content, and to the final approval of the version to be submitted.

Cecília L. de Oliveira: This author contributed to the acquisition of data, revising it critically for important intellectual content, and to the final approval of the version to be submitted.

Eliete Bouskela: This author contributed to the acquisition of data, revising it critically for important intellectual content, and to the final approval of the version to be submitted.

Maria Cristina C. Kuschnir: This author has contributed to the conception and design of the study, interpretation of data, drafting the article, revising it critically for important intellectual content and to the final approval of the version to be submitted.

Isabel R. Madeira: This author has contributed to the conception and design of the study, acquisition, analysis and interpretation of data, drafting the article, revising it critically for important intellectual content and to the final approval of the version to be submitted.

## Conflicts of interest

The authors declare no conflicts of interest.
